# Low health literacy is associated with patient–clinician discrepancy in the perceived severity of medical emergencies

**DOI:** 10.3389/fpubh.2025.1656755

**Published:** 2025-08-26

**Authors:** Matthias Nuernberger, Thomas Lehmann, Stefan Brodoehl, Jutta Huebner, Jan-Christoph Lewejohann

**Affiliations:** ^1^Department of Emergency Medicine, Jena University Hospital, Friedrich Schiller University, Jena, Germany; ^2^Department of Medical Statistics, Informatics and Data Science, Jena University Hospital, Friedrich Schiller University, Jena, Germany; ^3^Department of Neurology, Jena University Hospital, Friedrich Schiller University, Jena, Germany; ^4^Department of Internal Medicine, Jena University Hospital, Friedrich Schiller University, Jena, Germany

**Keywords:** health literacy, emergency medicine, emergency condition severity, public health, health seeking behavior, emergency department

## Abstract

**Introduction:**

Most adults in the United States and Europe have low health literacy (HL), which also has an impact on emergency care. It is unclear, whether low HL impairs the patients’ ability to evaluate the seriousness of their emergency and if it increases patient-clinician disagreement.

**Methods:**

In this prospective cross-sectional study in a German tertiary-care emergency department (ED), 257 adults (median age = 55 y) self-assessed the severity of their condition on arrival; simultaneously, an ED nurse and physician provided independent assessments. Thirty days later, an expert panel reviewed each case and issued a specialist evaluation. HL was assessed with the 16-item European Health Literacy Survey (HLS-EU-Q16) and categorized as adequate (*n* = 95), problematic (*n* = 119), or inadequate (*n* = 43). Three discrepancy indices were computed from age-, gender-, and education-adjusted assessments. Spearman correlations and Kruskal–Wallis tests compared agreement across HL levels; linear and logistic regressions examined predictors of discrepancy and severe outcome.

**Results:**

Patients with adequate HL showed the strongest alignment with clinicians (*ρ* = 0.24), whereas correlations were weaker in the inadequate group (ρ = 0.18). Discrepancies decreased as HL improved (*β* = −0.12 to −0.19, *p* < 0.05), but HL alone explained only up to 7% of variance. Concordant assessments increased with rising HL. Overestimation was most prevalent at inadequate HL level. In multivariable logistic modeling, each one-point increase in the Patient to Medical Team discrepancy raised the odds of a severe outcome by 27% (OR = 1.27, 95% CI [1.04, 1.55]), whereas each additional HL score point lowered the odds by 13% (OR = 0.87, 95% CI [0.77, 0.99]).

**Conclusion:**

Lower HL modestly but consistently enlarges the gap between patients’ and clinicians’ emergency assessments and is associated to a higher likelihood of a severe outcome via this mismatch. Although adequate HL improves agreement, half of these patients still struggle to evaluate severity. Routine teach-back communication might be helpful to identify discrepancies. These findings underscore the need for healthcare professionals to assess patients without prejudice, regardless of presenting symptoms, to ensure optimal medical care.

**Trial registration:**

Identifier, DRKS00032962.

## Introduction

Emergency departments (ED) are under sustained pressure. The United States reported 155 million visits in 2022 (47 per 100 residents) (1) while Germany recorded 12 million ambulatory ED presentations in 2023 (2)—the highest figure since national reporting began. Such large volumes routinely overwhelm capacity (3): a nationwide survey of 389 German EDs found 59% in active crowding (i.e., patient numbers exceed treatment capacities) on the index day, with 43% reporting severe or dangerous crowding (4). Systematic challenges like crowding or exit block (i.e., boarding of admitted patients in the ED in the absence of inpatient capacity) are linked to these volumes and compromise timely care and increases adverse events (5–10). A substantial share of attendances is clinically low acuity. Routine German ED data show that 33% of encounters did not require emergency treatment (11, 12). International reviews place the non-urgent proportion at 30–40% (12). Such cases consume resources, prolong waiting times for emergencies and drive costs that could be absorbed by primary care. Half of ED patients are self-referred (6), often influenced by an individual’s Health Literacy (HL) (13). Health literacy, the ability to locate, understand and apply health information, emerges therefore as a modifiable determinant in this regard (14, 15). The second German Health Literacy Survey found around 60% of adults have inadequate or problematic HL (16, 17) and even higher rates observed among ED patients (13); comparable US surveys indicate almost nine in 10 adults struggle with routine health tasks (18). Observational studies show patients with low HL make more frequent, and often non-urgent, ED visits than those with adequate HL (19–21). HL also shapes how people judge urgency. Lower self-reported HL can correlate with higher perceived treatment urgency, yet agreement between patient expectation and the triage category assigned by clinicians might be poor. An Australian study reported only 31% concordance (22). Misperception is amplified by health anxiety and the desire for rapid, comprehensive care, motives frequently cited by low-acuity ED users (23, 24). Age patterns add complexity: young adults, who typically score higher on functional HL tests, are nevertheless the group most likely to attend for non-urgent problems - more than three times as often as adults ≥65 years (25). Convenience, limited attachment to a general practitioner, and lifestyle considerations appear to outweigh the potential advantage conferred by higher HL (26, 27). Together, these findings suggest that limited HL inflates perceived urgency and encourages ED use, but that even adequate HL does not guarantee appropriate service choice when organizational or psychosocial factors intervene. Clarifying the interplay between HL and perceived versus actual severity is therefore crucial for designing measures that divert non-urgent cases without deterring those who need emergency care. The assessment of urgency is subjective and influenced by variables such as HL, leading to discrepancies between the perceptions of patients and healthcare professionals (22). For example, a broken toe may cause significant discomfort but does not constitute a medical emergency; conversely, a myocardial infarction may be accompanied by minimal symptoms but be life-threatening.

This study examines the ambiguous association between patients’ HL, their self-assessed emergency severity, the ED medical team’s assessment, and outcome in a high-volume German ED. The aim was to explore whether and how HL is associated with patients’ self-assessment of emergency condition severity.

## Research question

Does low health literacy impair the patient’s ability to assess the severity of their emergency condition? Does low health literacy lead to a high discrepancy between the patient’s self-assessment and the assessment of medical professionals? Does higher health literacy result in lower discrepancy in this regard?

## Methods

### Study design and population

This investigation was a prospective, monocentric, quantitative, analytic cross-sectional observational study conducted in the ED of a German university hospital. The aim was to investigate the influence of HL on patients’ subjective assessment of emergency condition severity. The study took place between November 21, 2023, and April 9, 2024. Written informed consent was acquired from all participants. The analytical cross-sectional science approach of this study was in compliance with the statement of Strengthening the Reporting of Observational Studies in Epidemiology (STROBE) (28). A pilot study was conducted prior to the main study to confirm feasibility (*n* = 10). The study was approved by the ethics committee of the Friedrich-Schiller-University Jena, Germany (Registration number: 2023-3106_1-Bef, date: 9^th^ October 2023) and preregistered on drks.de (DRKS00032962). All methods were carried out in accordance with relevant guidelines and regulations. All methods were carried out in compliance with the Declaration of Helsinki II.

All adult patients admitted to the ED of a German university hospital were eligible for inclusion. This ED handles approximately 37,000 visits per year. Sampling was performed daily during study hours, which alternated randomly between five time slots: 10–12 AM, 12 AM-2 PM, 2–4 PM, 4–6 PM, 6–8 PM. For further information regarding randomization see Supplementary 1. Patients were approached consecutively. 324 patients were included. The inclusion criteria required participants to be at least 18 years of age and classified under the Emergency Severity Index (ESI) triage category 2, 3, 4, or 5. Patients were excluded if they were admitted via emergency medical services or had a cognitive deficit that would impair their ability to complete the questionnaire. Of the 324 patients included, 257 patients had a complete data set. Only this data was used for analysis.

### Administration and study procedure

**Figure 1:**
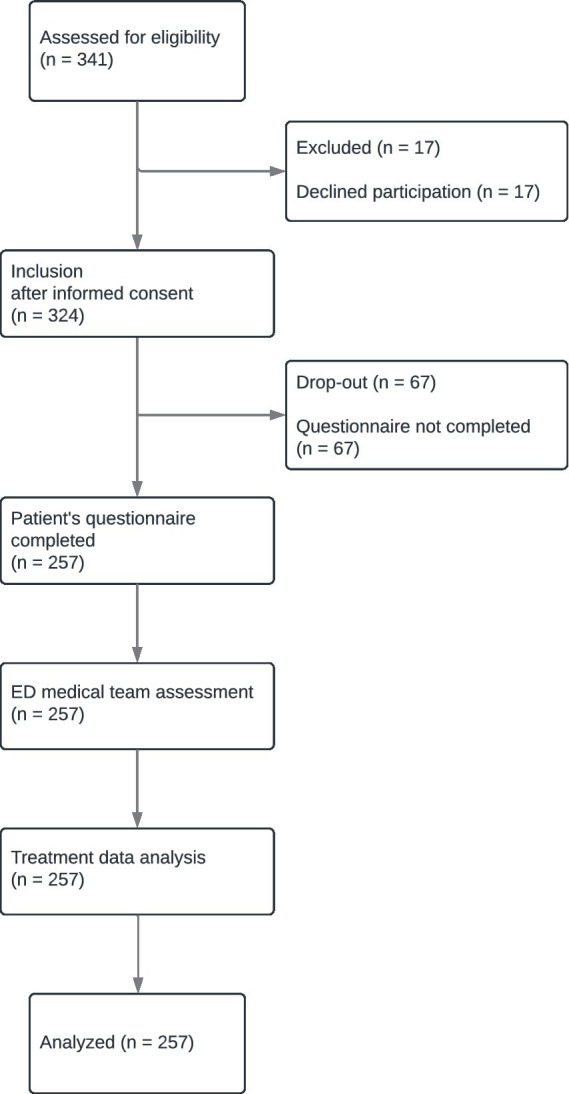
Data collection process [in concordance with STROBE (28)]. After informed consent had been obtained, included patients filled in the questionnaire. After the patient’s initial interaction with the medical personnel (ED nurse and physician), both professionals independently assessed the severity of the patient’s emergency condition. Subsequently, the actual condition severity was assessed by analyzing treatment data.

Upon admission and following the initial ESI triage process, patients were approached by the study team, including the principal investigator, to inform them about the study and invite their participation. After obtaining informed consent, patients were asked to complete the study questionnaire assessing their subjective perception of their emergency condition severity. This was done privately, in a quiet and separate room. Completion of the questionnaire took most patients around 5 min (range: 3–8 min). If a medical necessity arose during the data collection process, the process was terminated and not resumed. After the patient had completed their questionnaire, their ED nurse and physician were asked independently for their assessment of the emergency condition severity based on their first impression. The nurse and physician did not discuss this patient prior to their assessment. The composition of the medical team of physicians and nurses varied from shift to shift. If more than one interaction occurred between the medical team and the patient before the assessment, the data were excluded from the analysis to avoid mutual interference or influence by already collected test results. The final diagnosis of each patient and—if applicable—their treatment during their hospital stay was obtained through a retrospective examination of medical records 30 days post-admission performed by emergency medicine specialists (i.e., retrospective case assessment). See Figure 1 for a flowchart of the data collection process.

### Survey contents and outcome measures

#### Patient

The questionnaire for patients consisted of three sections. The first section consisted of questions addressing the patient’s emergency. The patient was asked to provide a subjective self-assessment of their emergency condition severity on an ordinal scale ranging from 1 (barely threatening) to 10 (life-threatening): “*How seriously ill or injured are you right now?*” (German: “Wie schwer krank oder verletzt sind Sie jetzt gerade?”).

Further questions were: “*How urgent do you think you should be seen by a doctor?”;* ranging from 0 (not urgent) to 10 (immediately).

“*Are you currently in pain? If yes: how severe is this pain on a scale from 0 to 10?”;* 0 (no pain) to 10 (unbearable extreme pain) on a numeric rating scale with visual depictions.

“*How much do your current symptoms restrict you in your everyday activities?”;* ranging from 0 (not restricted) to 10 (heavily restricted).

All results of the scales were added together and averaged to represent key symptom variants. This resulted in the patient’s assessment of their situation. Additional information can be found in Supplementary 2. This part of the questionnaire is similar to patient-reported outcome (PRO) measures like PROM-ED (29).

The second section of the questionnaire employed the German version of the European Health Literacy Survey’s 16 item questionnaire [HLS-EU-Q16 (30, 31)], a well-established and validated tool to assess health literacy in individuals developed by the Consortium of the European Health Literacy Project in their Health Literacy Survey (HLS-EU) project (32), see Supplementary 3. Its outcomes determine the individual’s HL level, which is then categorized into the HL groups inadequate HL, problematic HL, or adequate HL. Inadequate and problematic HL can be collectively be referred to as limited HL (30). Limited and low HL are commonly used interchangeably.

The third section asked the patients for their educational background (secondary school, high school, college, university) and age. College and university are different educational paths in Germany.

#### ED medical team

The ED medical team subjectively evaluated the severity of the emergency on presentation. Junior ED physicians in training (ED physicians, *n* = 11) assessed the patient. They had an average working experience of 1.4 years, ranging from 1 to 3 years. ED nurses (*n* = 15) also assessed the patient at presentation as well. They had an average working experience of 8.9 years, ranging from 4 to 12 years.

Immediately after their initial contact with the patient, the ED medical team (ED nurse and physician) was asked independently for their subjective assessment of the patient’s condition severity on an ordinal scale: “*How seriously ill or injured is this patient right now?*.” This assessment also ranged from 1 (barely threatening) to 10 (life-threatening). The duration of the team’s interaction with the patient was recorded in minutes, as well as their professional experience in years. Any exchange between personnel prior to answering the questions was avoided.

#### Case assessment by emergency medicine specialists

Four emergency medicine specialists analyzed the patient cases 30 days after their presentation based on the medical records in the clinical information system. They had an average working experience of 10.2 years, ranging from 6 to 15 years. Only data from the day of ED presentation to 30 days post-presentation was included. They worked independently and were blinded to potentially influential contextual factors (e.g., presentation time or triage). Agreement rate between the emergency medicine specialists was 92%. Their subjective case assessment was mapped on an ordinal scale from 1 (barely threatening) to 10 (life-threatening) which represents the severity of the emergency in retrospect. 10 signifies an initially immediately life-threatening situation. Their chart-based assessment was created under consideration of observer-reported (ObsRO) and clinician-reported outcome (ClinRO) measures. Supplementary 3 contains examples of diagnosis severity assessments. This retrospective analysis aligned with expected outcomes, with high severity (≥ 5.59, i.e., *Mean* + 1 *SD*) observed in 14.4% (*n* = 37) of cases. We defined this Severe Outcome as a specialists’ assessment ≥ 1 SD above the cohort mean, a distribution-based threshold that selects the upper 15% of presentations. Most triage systems such as the Emergency Severity Index in our ED reserve their highest two categories for approximately the top decile-to-quintile of acuity, mirroring an M + 1 SD cut-point (33, 34). Stricter cut-offs (≥ 2 SD, 2% of cases) yield too few events for reliable multivariable models, whereas looser median splits dilute clinical signal. Prior HL studies have validated analogous upper-quartile or ≥ + 1 SD rules as markers of high risk (35, 36). Clinically, this manifested itself in our study population in the form of monitoring requirements, inpatient admission, surgical procedures, invasive interventions, complications, or a combination of these factors. Therefore, a score higher than or equal to 6 is considered to point to a condition permanently dangerous for the patient’s health, as it reflects conditions exceeding the average severity (3.65) by one standard deviation (1.94) and is thus referred to as ‘Severe Outcome’.

#### Discrepancy

Discrepancy describes the difference between the patient’s assessment and the assessment made by the ED medical team and the severity indicated by emergency medicine specialists’ assessments. A greater positive or negative distance from 0 indicates a greater divergence, suggesting a reduced predictive accuracy of the patient’s self-assessment. Negative values below −1 represent an underestimation by the patient regarding their condition severity, hence called “Negative Discrepancy.” Positive values above 1 point to a deviation, in which the patient’s assessment surpasses the assessments by the medical professionals, hence referred to as “Positive Discrepancy.” Values within the range of −1 and 1 were considered to reflect “Concordance” between assessments and were therefore labeled as such instead of discrepancy. For statistical analysis, absolute values were used to determine the degree of dis−/agreement, and actual values were used to determine the direction of dis−/agreement.

Three variants of discrepancy were used: Discrepancy (All), which includes all assessments, Discrepancy (Medical Team), which includes only the prospective assessments of the patient and the ED team, and Discrepancy (Specialists), which includes the assessments of the patients and the retrospective assessments of the case by the emergency medicine specialists. In this way, the different points in time and the level of knowledge about the case are considered. To calculate the discrepancy, the average of the assessments of the ED physicians, the ED nurses and the emergency medicine specialists in various combinations was subtracted from the patients’ assessments as a mean value:


$$\begin{array}{l} \text{Discrepancy}\;(\mathrm{All})=\text{Patient}–\\ {}[\text{Mean}\;(\mathrm{ED}\;\text{Physician},\mathrm{ED}\;\text{Nurse},\text{Emergency medicine specialists})]. \end{array}$$

Discrepancy(Medical Team)=Patient–[Mean(EDPhysician,EDNurse)].

Discrepancy(Specialists)=Patient–Emergency medicine specialists.

### Data analysis

The minimum required sample size was calculated to be 248 patients (see Supplementary 4 for details).

All data were analyzed using descriptive methods. Normality was assessed using the Shapiro–Wilk test and Q-Q plots. Spearman rank-order correlations (*ρ*) quantified how closely assessments matched. Fisher’s *z* values indexed effect size. Discrepancy was contrasted across HL levels with Kruskal–Wallis tests; significant omnibus results were followed by Dunn post-hoc contrasts with Bonferroni correction, and effect size was expressed as eta-squared (η^2^). Multiple OLS regressions predicted each Discrepancy from HL score, age, education, and gender, reporting unstandardized Bs, SEs, and *R*^2^. See Supplementary 5 for a detailed description. All statistical analyses were performed using SPSS Statistics version 29.0.0 build 241 (IBM, New York, United States) and in collaboration with a faculty biostatistician who remained blinded to the study conditions. Figures were created using Prism version 10.2.1 build 339 (GraphPad Software, LLC., Boston, United States). All results were evaluated at a two-sided significance level of *α* = 0.05, unless otherwise specified.

## Results

This study addresses the question of whether low HL impairs a patient’s ability to assess the severity of their emergency condition and whether low HL can lead to a high discrepancy between the patient’s self-assessment and the assessment of medical professionals. Furthermore, this study investigates if higher HL results in lower discrepancy in this regard.

### Characteristics of participants

341 patient cases were screened upon admission. 17 screened patients declined participation. 324 patients were included. Data of 67 patients (21%) was later omitted due to incomplete HL questionnaires, which occurred due to organizational processes (e.g., call for X-ray). 14 of those excluded patients had other missing data, mainly the physician’s or nurse’s assessment. Data from 257 patient cases was analyzed, see Figure 1. Only complete data sets were analyzed.

The age of included patients followed the usual distribution for our ED. Mean age of participants was 53 years (*Md* 55, *SD* 22.05, range: 19–93). Most patients included reported to have completed high school (42%).

Patients were stratified into three groups based on their HL level as determined by HLS-EU-Q16: adequate (*n* = 95), problematic (*n* = 119), and inadequate (*n* = 43) HL. Problematic and inadequate HL were collectively referred to as the limited HL group (30). 63% of the patients included scored in the inadequate or problematic HL categories. There were no demographic differences between the groups. See Table 1 for a detailed group description.

**Table 1:** HL group characteristics.

	HL inadequate (*N* = 43)	HL problematic (*N* = 119)	HL adequate (*N* = 95)
Gender			
Female	44% (19)	53% (63)	51% (48)
Male	56% (24)	47% (56)	49% (47)
Age			
in years	56.84 (22.52)	52.50 (22.46)	51.97 (21.35)
Education			
Secondary School	12% (5)	10% (12)	16% (15)
High School	46% (20)	40% (48)	27% (26)
College	21% (9)	24% (29)	25% (24)
University	21% (9)	25% (30)	32% (30)
Admission			
Self	44% (19)	44% (52)	33% (31)
Family or Friends	7% (3)	10% (12)	14% (13)
Physician	44% (19)	38% (45)	41% (39)
Employer	4% (2)	8% (10)	13% (12)
Condition severity assessment			
Patient	5.39 (1.65)	5.70 (1.81)	4.79 (1.69)
adjusted	5.43 (1.57)	5.64 (1.76)	4.89 (1.71)
ED Nurse	4.76 (1.16)	4.22 (1.54)	4.21 (1.63)
adjusted	4.65 (1.17)	4.19 (1.53)	4.21 (1.54)
ED Physician	4.00 (1.53)	4.05 (1.69)	3.84 (1.61)
adjusted	3.87 (1.47)	4.03 (1.65)	3.87 (1.58)
Chart-based case assessment			
Specialists	3.79 (2.08)	3.60 (2.10)	3.68 (1.88)
adjusted	3.72 (2.16)	3.58 (1.93)	3.73 (1.83)
Time to the ED			
10–12 AM	12% (5)	11% (13)	12% (11)
12 AM-2 PM	12% (5)	12% (14)	15% (14)
2–4 PM	16% (7)	17% (20)	20% (19)
4–6 PM	28% (12)	29% (35)	27% (26)
6–8 PM	32% (14)	31% (37)	26% (25)
Weekday to the ED			
Monday	9% (4)	8% (10)	11% (10)
Tuesday	7% (3)	8% (9)	7% (7)
Wednesday	14% (6)	16% (19)	15% (14)
Thursday	7% (3)	7% (8)	7% (7)
Friday	14% (6)	15% (18)	14% (13)
Saturday	26% (11)	22% (26)	23% (22)
Sunday	23% (10)	24% (29)	23% (22)

Values presented as percentage (count). Emergency condition severity assessments by Patient, ED Nurse, and ED Physician, categorized by HL group; mean (SD) and adjusted marginal mean (SD). Chart-based retrospective case assessment by emergency medicine specialists; mean (SD) and adjusted marginal mean (SD). Adjusted marginal means include correction for age, education, and gender. HL, Health literacy; ED, Emergency Department.

Group characteristics are described in Table 1. The overall distribution of diagnoses in the included patients was representative of the common reasons for presentation to our ED, Supplementary 6, 7.

The duration of initial contact between patient and ED physician was 3–6 min, while it was 9–12 min for ED nurses.

### Emergency condition severity assessments

Assessments were covariate-adjusted (Age, Education, and Gender) for analysis, see Supplementary 8. Only the regression analysis was calculated using unadjusted values. Mean severity assessment by the patients across the whole sample was 5.33 (*Md* 5.42, *SD* 1.74), while it was 4.27 (*Md* 4.54, *SD* 1.48) for the ED nurses, 3.95 (*Md* 3.70, *SD* 1.59) for the ED physicians, and 3.66 (*Md* 3.77, *SD* 1.93) for the emergency medicine specialists.

### HL level comparison

Assessments were compared between HL levels, Table 2. Only the severity rated by the patients differed statistically significant between the HL levels, indicating an association between self-assessment and HL.

**Table 2:** Adjusted scores were revisualized for age, education, and gender (grand-mean centered).

Assessment	Inadequate HL	Problematic HL	Adequate HL	*F*	*df*	*p*
Patient	5.43 (1.57)	5.64 (1.76)	4.89 (1.71)	6.19	(2, 246)	0.002
ED Nurse	4.65 (1.17)	4.19 (1.53)	4.21 (1.54)	1.26	(2, 197)	0.285
ED Physician	3.87 (1.47)	4.03 (1.65)	3.87 (1.58)	0.27	(2, 212)	0.762
Specialists	3.72 (2.16)	3.58 (1.93)	3.73 (1.83)	0.16	(2, 221)	0.849

Higher values indicate greater perceived severity; mean (SD). HL levels were compared using Kruskal-Wallis-test. HL, Health literacy; ED, Emergency Department.

### Correlation analysis

Correlation analysis was performed for all assessments and between HL levels, see Table 3. Across the full sample, all severity assessments except patients’ and specialists’ were positively correlated (Spearman’s *ρ* = 0.18–0.34). Patient self-assessments showed a small association with ED nurses’, ρ = 0.20, *p* < 0.01, and ED physicians’ (ρ = 0.18, *p* = 0.01). Clinician assessments were interrelated: nurse–physician, *ρ* = 0.24, *p* < 0.001; physician–specialist, *ρ* = 0.44, *p* < 0.001; and nurse–specialist, ρ = 0.22, *p* < 0.01.

**Table 3:** Correlation analysis.

Overall	Patient	ED nurse	ED physician	Specialists
Patient		< 0.01	< 0.01	0.702
ED Nurse	0.20*		< 0.001	< 0.01
ED Physician	0.18*	0.24*		< 0.001
Specialists	−0.03	0.22*	0.34*	
Adequate HL				
Patient		< 0.01	0.15	0.32
ED Nurse	0.30*		< 0.01	0.16
ED Physician	0.16	0.39*		< 0.001
Specialists	0.11	0.18	0.40*	
Problematic HL				
Patient		0.24	< 0.01	0.30
ED Nurse	0.12		0.26	0.09
ED Physician	0.25*	0.12		< 0.01
Specialists	−0.10	0.18	0.29*	
Inadequate HL				
Patient		0.33	0.99	0.45
ED Nurse	0.18		0.08	< 0.01
ED Physician	0.00	0.33		0.04
Specialists	−0.12	0.44*	0.36*	

All coefficients are two-tailed Spearman *ρ*. *Asterisk and bold values mark significant correlations (*p* < 0.01). Upper right corner depicts *p*, lower left corner depicts Spearman ρ. HL, Health literacy; ED, Emergency Department.

### Correlation analysis in HL groups

#### Adequate HL

Patient assessments correlated with nurses’, *ρ* = 0.30, *p* = 0.006. Inter-clinician associations were stronger: nurse–physician, *ρ* = 0.39, *p* = 0.001, and physician–specialist, ρ = 0.40, *p* < 0.001. The remaining correlations were nonsignificant, *p* > 0.05.

#### Problematic HL

Patient assessments correlated modestly with physicians’, ρ = 0.25, *p* = 0.006, and physician assessments correlated with specialists’, ρ = 0.29, *p* = 0.002. No other coefficients reached significance, *p* > 0.05.

#### Inadequate HL

Only clinician–clinician associations reached significance: nurse–specialist, ρ = 0.44, *p* = 0.006, and physician–specialist, ρ = 0.36, *p* = 0.047. All patient–clinician correlations were nonsignificant, *p* > 0.05.

### Regression analysis

A binary logistic regression examined whether a Severe Outcome (specialists’ assessment > 5.59) could be predicted using patient-related data. The overall model was significant, R^2^ = 0.09, *N* = 249. Controlling for age, education, and gender, higher HL test (HLS-EU-Q16) scores were associated with lower odds of a severe outcome, OR = 0.84, 95% CI [0.75, 0.93], *p* = 0.001. Conversely, higher patient self-assessments increased the odds, OR = 1.21, 95% CI [1.07, 1.37], *p* = 0.003. Greater educational attainment was also protective, OR = 0.83, 95% CI [0.70, 0.98], *p* = 0.025. Age and gender were non-significant (*p* ≥ 0.23). Thus, both HL and the patient’s own severity perception meaningfully contribute to predicting whether a specialist will judge a case as severe. The models explained 10% of the variance in the Severe Outcome variable, indicating relevant predictive value, see Supplementary 9.

Discrepancy between patients’ and clinicians’ assessments in all variations averaged between 1.5 and 2 in most cases, see Table 4.

**Table 4:** Discrepancy between patient’s and medical team’s and/or emergency medicine specialists’ assessments in three variations: all (including all assessments), Medical Team (including Patient, ED Nurse, and ED Physician), and Specialists (including Patient and Specialists), mean (SD).

Discrepancy	Overall	Inadequate HL	Problematic HL	Adequate HL	Statistics
All*	1.51 (2.03)	1.48 (2.15)	1.84 (2.09)	1.09 (1.84)	*H* (2) = 7.07 *p* = 0.029
All* (absolute)	2.08 (1.44)	2.02 (1.60)	2.34 (1.46)	1.63 (1.28)	*H* (2) = 7.76 *p* = 0.021
Medical team*	1.33 (1.96)	1.21 (2.08)	1.64 (1.98)	0.98 (1.84)	*H* (2) = 7.02 *p* = 0.030
Medical team* (absolute)	1.98 (1.30)	1.86 (1.46)	2.15 (1.34)	1.62 (1.24)	*H* (2) = 6.92 *p* = 0.031
Specialists	1.75 (2.64)	1.81 (2.86)	2.11 (2.74)	1.26 (2.31)	*H* (2) = 4.23 *p* = 0.121
Specialists (absolute)	2.55 (1.96)	2.57 (2.22)	2.90 (2.09)	2.15 (1.78)	*H* (2) = 4.12 *p* = 0.127

*Asterisk marks significant comparisons. HL, Health literacy; ED, Emergency department.

### Group comparison

The size of the discrepancy between patients’ self-assessments and clinicians’ assessments depended on HL level. For Discrepancy All, the difference across inadequate-, problematic-, and adequate-HL groups was significant, *H* (2) = 7.07, *p* = 0.029, ε^2^ = 0.02, indicating a small effect. Post-hoc tests revealed that adequate-HL patients exhibited smaller gaps than both inadequate-HL (*p* = 0.011) and problematic-HL patients (*p* = 0.021), whereas the latter two groups did not differ (*p* = 0.83). The same pattern emerged for Discrepancy Medical Team, *H* (2) = 7.02, *p* = 0.030, ε^2^ = 0.02; adequate-HL patients differed from inadequate (*p* = 0.014) and problematic (*p* = 0.027) patients, while inadequate and problematic groups were similar (*p* = 0.79). By contrast, the Discrepancy Specialists did not vary by HL level, *H* (2) = 4.23, *p* = 0.121, ε^2^ = 0.01. Thus, lower health literacy is associated with modestly larger mismatches between patient and nurse/physician assessments, but not between patient and specialist assessments.

**Figure 2:**
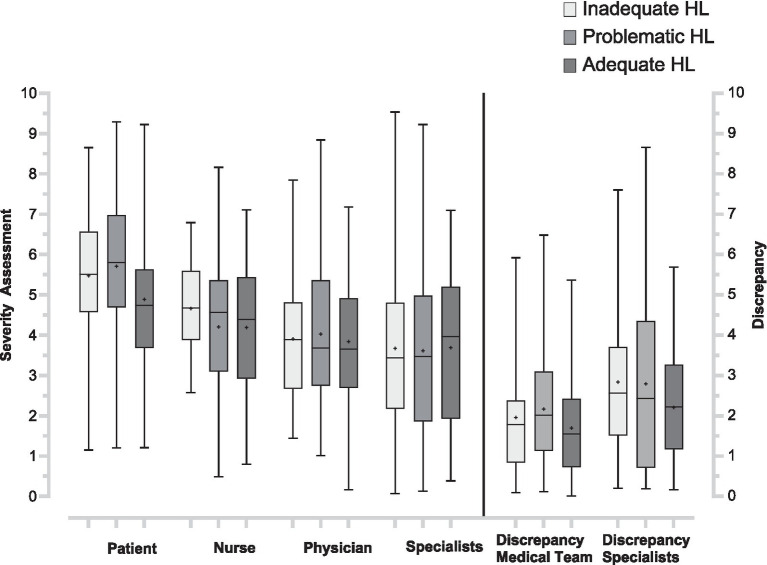
Health literacy group comparison for outcome measures and discrepancies. The box plots (min-max) on the left show condition severity assessments (Patient, ED Nurse, ED Physician, and emergency medicine specialists), range 0–10. The box plots (min-max) on the right show discrepancies between patients’ and staff assessments (Discrepancy Medical Team, Discrepancy Specialists). Mean values are marked in the box plots (+). HL, Health literacy; ED, Emergency Department.

Using absolute values to index the *magnitude* of disagreement (ignoring direction) yielded very similar results. Discrepancy All still differed across health-literacy groups, *H* (2) = 7.76, *p* = 0.021, ε^2^ = 0.02, indicating a small effect. Post-hoc contrasts confirmed that adequate-HL patients disagreed less with the full clinical team than did both inadequate-HL (*p* = 0.013) and problematic-HL patients (*p* = 0.025); the latter two groups did not differ (*p* = 0.77). An analogous pattern emerged for Discrepancy Medical Team, *H* (2) = 6.92, *p* = 0.031, ε^2^ = 0.02, with the adequate-HL group again showing smaller mismatches than the other two (*p* < 0.05), see Figure 2. In contrast, Discrepancy Specialists did not vary by HL level, *H* (2) = 4.12, *p* = 0.127, ε^2^ = 0.01. Thus, even when only the size of the gap is considered, lower HL remains associated with larger patient–clinician disagreements for nurses and physicians, whereas agreement with specialists is unaffected by HL level.

### Regression analysis

Multivariable regression analysis was performed to evaluate the relationship between HL, discrepancy, and the covariates age, education, and gender, Supplementary 10. The numerical value of the patient’s HLS-EU-Q16 score was used for HL.

Controlling for age, education, and gender, higher HL scores reliably predicted smaller patient–clinician mismatches. For Discrepancy All, HL was a significant negative predictor, *b* = −0.15, *t*(240) = −2.63, *p* = 0.009, explaining 4% of the variance (*R*^2^ = 0.04). A comparable, marginally significant relation appeared for Discrepancy Medical Team (*b* = −0.11, *t*(229) = −1.84, *p* = 0.067, *R*^2^ = 0.02), and the association was strongest for Discrepancy Specialists (*b* = −0.23, *t*(219) = −3.83, *p* < 0.001, *R*^2^ = 0.07).

Age and education did not affect any discrepancy indices except Discrepancy Specialists, where older and less-educated patients showed greater results (age: *p* = 0.022; education: *p* = 0.008). Gender was unrelated to all three outcomes. In sum, higher HL scores consistently correspond to smaller patient–clinician disagreements, particularly with specialists, while demographic factors add little explanatory power. The models accounted for only 2–7% of the variance, implying that HL is only partly accounting for patient–clinician incongruence.

Concordance was most prevalent among patients with adequate HL for Discrepancy All (34%) and Medical Team (33%), see Table 5. For Discrepancy with the Specialists, the problematic HL group achieved a slightly higher prevalence of concordance (26 vs. 22%). Positive discrepancy was around 10% more frequent in inadequate and problematic HL patients than in adequate HL patients. Negative discrepancy was slightly more frequent in inadequate HL patients compared to the other groups. The combination of negative discrepancy and severe outcome was frequent.

**Table 5:** Group characteristics regarding discrepancy.

Discrepancy	Inadequate HL; *n* = 43			Problematic HL; *n* = 119			Adequate HL; *n* = 95		
	=	+	–	=	+	–	=	+	–
All	19%	65%	**16%**	25%	**66%**	9%	**34%**	53%	13%
Medical Team	25%	60%	**15%**	22%	**67%**	11%	**33%**	55%	11%
Specialists	7%	**74%**	**19%**	**26%**	62%	12%	22%	60%	18%
Severe outcome	of total: 5 (12%)			of total: 18 (15%)			of total: 14 (15%)		
All	2	0	3	7	7	4	7	1	6
Medical Team	2	0	3	4	12	3	7	4	4
Specialists	0	0	5	10	2	8	4	1	11

Discrepancy describes the deviation between the patient’s assessment and the combined assessment of ED nurses, ED physicians, and emergency medicine specialists in three different combinations: Discrepancy All, Discrepancy Medical Team and Discrepancy Specialists. Bold values indicate the most frequent feature within this group; presented as percentage of group. = Equal indicates Concordance, + Plus indicates Positive Discrepancy, and – Minus indicates Negative Discrepancy. Severe Outcome of total describes the proportion of severe cases (Specialists’ assessment > 5.59) as percentage on this HL level. Each cell below shows the number of Severe Outcome within that discrepancy category, the HL level, and the direction of discrepancy. HL, Health literacy; ED, Emergency department.

Severe cases were as expected: 12–15%. The dangerous combination of Severe Outcome and negative discrepancy (indicative of patients underestimating their symptoms) was not rare (3–11%) and prevalent on all HL levels yet emphasized for inadequate HL.

### Patient outcome measures

In the multivariable logistic model, a Discrepancy Medical Team independently increased the likelihood of a Severe Outcome: each one-unit rise was associated with 30% higher odds, OR = 1.30, 95% CI [1.07, 1.59], *p* = 0.008, after controlling for age, gender, and education. Higher nurse (OR = 1.20, 95% CI [1.01, 1.43], *p* = 0.037) and physician assessments (OR = 1.22, 95% CI [1.01, 1.47], *p* = 0.040) provided comparable incremental risk, whereas a higher health-literacy score lowered the odds by 12% (OR = 0.88, 95% CI [0.78, 0.99], *p* = 0.028). Age showed a marginal protective trend, *p* = 0.063, and neither patient self-assessments nor education reached significance. The model accounted for 24% of the variance in the severe-outcome indicator (*R*^2^ = 0.24).

## Discussion

The present findings help to understand how HL limitations modulate patients’ appraisal of emergency severity. Consistent with our hypothesis, patient–clinician agreement declined stepwise from adequate to problematic to inadequate HL. In bivariate terms, the correlation between patients’ assessment and the composite clinician evaluation was modest yet larger in the adequate-HL group (*ρ* = 0.24) than in the inadequate-HL group (ρ = 0.18). Multivariable residual models reinforced this pattern: after controlling for age, education, and gender, higher HL scores predicted smaller discrepancies for the full clinical team, for the on-site ED medical team, and most strongly for the retrospective specialist’s evaluation (*β* = −0.12 to −0.19). Absolute discrepancies likewise differed by HL level (η^2^ ≈ 0.02). Nonetheless, HL alone explained only up to 7% of the variance, indicating that it is a contributory rather than dominant source of incongruence. Additionally, lower HL also produces *larger* disagreements: concordance fell from 32% in the adequate group to 24 and 19% in the problematic and inadequate HL levels, while positive discrepancies rose from 53% to roughly two-thirds (65–66%). Negative discrepancies occurred least often but were proportionally greatest in inadequate HL (16%). Thus, lower HL is characterized by more frequent over-estimation and occasional, potentially dangerous, under-estimation of urgency. Linking this mismatch to clinical endpoint, each additional point on the HLS-EU-Q16 reduced the odds of a severe outcome by 13%, whereas every one-unit increase in the Medical-Team discrepancy raised those odds by 27%. Lower HL can therefore inflate both the frequency and the magnitude of patient–clinician disagreement. However, the effect size remains modest. Even with adequate HL, half of the patients over- or under-estimated their condition. Younger, highly educated, and most likely adequately health literate patients may minimize symptoms due to illness-related threat appraisals, whereas the least-literate patients may delay or misdirect help-seeking. Consequently, HL cannot be the sole triage criterion. Rather, the *discrepancy* itself emerges as an actionable marker: large gaps between patient and medical professionals signaled the highest risk irrespective of literacy level. Together, these findings suggest that HL is a meaningful yet modest modifier of patient–clinician agreement rather than a dominant determinant. Because neither clinicians nor patients can reliably estimate each other’s informational needs, defaulting to the assumption that all patients struggle to appraise emergency symptoms may be prudent. Systematic HL screening in the ED has not improved outcomes and remains impractical (37–39). Instead, surrogate indicators like age and brief teach-back techniques may offer efficient risk stratification. Ultimately, accurate triage still rests on professional assessment, but enhancing symptom-interpretation skills particularly in patients with lower HL should remain a parallel goal to strengthen patient safety. The present findings accord well with recent research that links limited HL to patient-clinician discordance (22). In a recent multicenter German survey, lower HL was independently associated with judging low-acuity presentations as highly urgent, whereas adequate HL patients showed closer alignment with nurse triage categories (24). An Italian study found that 24% of low HL callers of emergency medical services *under*estimated urgency, arriving with conditions nurses deemed life-threatening (40), mirroring our results. Norwegian primary-care EDs also show a similar bidirectional mismatch: only 38% of walk-in patients agreed with physicians on urgency, and discordance was greatest among those with lower education, a robust HL proxy (41). Our data also echoes studies showing that this mismatch can be clinically consequential (40, 42). US studies tie patient-clinician disagreement on illness severity to higher admission risk and resource use (43), while a Dutch cohort linked inadequate HL to delayed myocardial-infarction presentation and worse outcomes (44). Yet, HL still explained only a share of the variance, underscoring that it is only contributory. Psychological factors like health anxiety (45) and structural barriers like primary-care access (24) might account for the residual mismatch, as suggested by surveys of non-urgent ED users (26). Overall, the findings position HL as a threshold competence that should be further developed and supported: adequate HL reduces large over-estimations and improves concordance but does not eliminate misjudgment. Discrepancy magnitude itself might be a sensitive real-time marker of clinical risk. Interventions should therefore combine universal, literacy-adapted communication (e.g., plain language, teach-back, visual aids) with discrepancy-triggered safety mechanisms (e.g., alerting senior staff when patient’s and physician’s evaluation diverge). Such strategies recognize the modest but nevertheless feasible role of HL while at the same time utilizing the signal inherent in the agreement or disagreement between patient and medical team.

### Limitation

This study is not devoid of limitations: Clinical outcomes were only partially included in our results as they are part of the retrospective case assessment by the emergency medicine specialists and in the form of the variable Severe Outcome. Regardless of this, no delays, diagnostic errors, or adverse events were measured. These aspects are significant but are not part of our research question. We compared self-reported severity in the context of different HL levels, subjective assessments, and retrospective chart-based evaluations. Therefore, our data cannot be used to draw conclusions about the direct impact of HL on emergency medical care. In general, subjective evaluations inherently carry the risk of bias. However, since the professional assessments were conducted by a specialized team, biases should have been minimized. As these biases (e.g., confirmation or anchoring bias) are part of subjective assessments and opinion formation, they are ultimately unavoidable and to a certain extent necessary. For the chart-based retrospective analysis the specialists worked independently and were blinded to potentially influential contextual factors (e.g., presentation time or triage). The study population was limited to patients seen during daytime hours to achieve broad coverage and feasibility of the procedure. However, this approach may have introduced some degree of inclusion bias, as typical nocturnal emergencies such as myocardial infarction or intoxication were inevitably less prevalent. However, the most prevalent emergency conditions are represented and the cases included cover a broad time period. In addition, the groups are consistent with other surveys in terms of gender, age, HL, education, and time as well as day of presentation to the ED (5, 16). HL was assessed using the HLS-EU-16 self-assessment questionnaire. Self-assessments, especially those based on questionnaires, are susceptible to generalized over- or underestimation. Despite these limitations, this questionnaire was employed because it is a validated scientific instrument, and it is suitable for a fast-paced setting such as the ED. As nearly all tools for evaluating HL are based on self-assessment, no more suitable alternative was identified for this trial. Even though some questionnaire items have been debated in the literature (46), these were deemed irrelevant for the subject of this trial.

During data collection, a high number of participants (21%) did not complete their questionnaire in full and therefore had to be excluded, as there was no plan to complete the questionnaire later. In all cases, this was due to organizational procedures in the emergency department (e.g., call-up for X-ray examination). No case had to be excluded for medical reasons. Ideally, obtaining subjective assessments of condition severity from patients before triage could help lower the procedural bias and the potential for unconscious influence. However, this was not feasible within the constraints of routine ED operations.

## Conclusion

Low HL modestly but consistently impairs patients’ accuracy in judging emergency severity, leading to larger and more frequent disagreements with clinicians. Although higher HL improves concordance, it does not fully protect against misjudgment. Discrepancy between patient and medical team proved the most sensitive predictor of severe outcomes, suggesting that algorithms combining HL-adapted communication with discrepancy-based risk flags may best enhance patient safety.

## Data Availability

The raw data supporting the conclusions of this article will be made available by the authors, without undue reservation.
