# Supplementary material for: Low health literacy is associated with patient–clinician discrepancy in the perceived severity of medical emergencies
Source: Front Public Health. 2025 Aug 26;13:1656755. doi: 10.3389/fpubh.2025.1656755 (PMC12417502; doi:10.3389/fpubh.2025.1656755)
Supplement: Supplementary file 1 [file Supplementary_file_1.docx]

# Supplementary

## Randomization of sampling time slots:

Patient cases were included daily during study hours, which alternated randomly between five time slots: 10-12 AM, 12 AM-2 PM, 2-4 PM, 4-6 PM, 6-8 PM. The randomization for these time slots was implemented using permuted block randomization with dice rolls resulting in a predefined schedule and equally distributed time slots.

## Patient questionnaire – additional questions:

“*How long have you been suffering from your current symptoms?”;* less than 24 hours, 24 hours until 2 days, 2 days until one week, 1 week or longer.

Additionally, the type of admission (self, family or friends, physician, or employer) was asked.

## HLS-EU-Q16

The administration and analysis of HLS-EU-Q16 followed established guidelines and best practices (1,2). Permission for its use was obtained. If two or more answers were missing, the questionnaire was deemed incomplete, and this patient’s data excluded from analysis.

The HLS-EU-Q16 is a shortened of the HLS-EU-Q47, which was developed by Sørensen et al. in the Consortium of the European Health Literacy Project in 2009 (1) and first used in the European Health Literacy Survey (HLS-EU) in 2011(3). It is intended to measure a person's health literacy through self-assessment. All HLS-EU questionnaires are based on the integrated model of health literacy as proposed by Sørensen et al. (4). The HLS-EU-Q16 was developed inside the Consortium of the European Health Literacy Project using data from the first HLS-EU (5,6). In this study, we used the German version of the HLS-EU-Q16 questionnaire, see *Table 1*.

The 16 items of the HLS-EU-Q16 were selected so that they represent the matrix of the original 47 items as consistently as possible and at the same time exhibit the best possible psychometrics. The selection was made using a single-parameter dichotomous Rasch model and Item Response Theory(5,7). They cover all three areas of health literacy that are examined in the HLS-EU-Q47: Health Care (question 1-7), Disease Prevention (question 8-12), and Health Promotion (question 13-16). They also cover all four dimensions of health-related information management: accessing and obtaining information, understanding information, processing information and applying information. Only the “use of information” aspect of “health promotion” is not covered in HLS-EU-Q16 compared to HLS-EU-Q47, as none of the original items met the Rasch criteria. During validation, the correlation of HLS-EU-Q16 results with results from HLS-EU-Q47 were very high (mean *r* = 0.82, range *r* = 0.73-0.88 for different language versions).

In addition to reducing the items, the four possible answers (very easy, rather easy, rather difficult, very difficult) are merged into two categories (easy, difficult) during analysis, resulting in a dichotomous (0, 1) sum score and therefore level allocation: inadequate HL (0-8 points), problematic HL (9-12 points), and adequate HL (13-16 points). Inadequate and problematic HL can be combined as limited HL.

We chose the HLS-EU-Q16 because its short application time of about 3 to 5 minutes makes it well suited for the dynamic environment of an ED. At the same time, the HLS-EU-Q16 is a widely used and generally recognized tool for assessing HL in individuals(8). Naturally, the results of HLS-EU-Q16 are less robust compared to HLS-EU-Q47: it shows an early ceiling effect for higher HL levels (8), but this is not detrimental for our investigation. Higher HL is not separated into sufficient and excellent HL in the HLS-EU-Q16 but is summarized as adequate HL.

| Nr | **Question** | **Domain** |
| --- | --- | --- |
|  | Auf einer Skala von sehr einfach bis sehr schwierig:  Wie einfach ist es Ihrer Meinung nach… |  |
| 1 | …Informationen über Therapien für Krankheiten, die Sie betreffen, zu finden? | **Health Care** |
| 2 | …herauszufinden, wo Sie professionelle Hilfe erhalten, wenn Sie krank sind? |  |
| 3 | …zu verstehen, was Ihr Arzt Ihnen sagt? |  |
| 4 | …die Anweisungen Ihres Arztes oder Apothekers zur Einnahme der verschriebenen Medikamente zu verstehen? |  |
| 5 | …zu beurteilen, wann Sie eine zweite Meinung von einem anderen Arzt einholen sollten? |  |
| 6 | …mit Hilfe der Informationen, die Ihnen der Arzt gibt, Entscheidungen bezüglich Ihrer Krankheit zu treffen? |  |
| 7 | …den Anweisungen Ihres Arztes oder Apothekers zu folgen? |  |
| 8 | …Informationen über Unterstützungsmöglichkeiten bei psychischen Problemen, wie Stress oder Depression, zu finden? | **Disease**  **Prevention** |
| 9 | …Gesundheitswarnungen vor Verhaltensweisen wie Rauchen, wenig Bewegung oder übermäßiges Trinken zu verstehen? |  |
| 10 | …zu verstehen, warum Sie Vorsorgeuntersuchungen brauchen? |  |
| 11 | …zu beurteilen, ob die Informationen über Gesundheitsrisiken in den Medien vertrauenswürdig sind? |  |
| 12 | …aufgrund von Informationen aus den Medien zu entscheiden, wie Sie sich vor Krankheiten schützen können? |  |
| 13 | …Informationen über Verhaltensweisen zu finden, die gut für Ihr psychisches Wohlbefinden sind? | **Health**  **Promotion** |
| 14 | …Gesundheitsratschläge von Familienmitgliedern oder Freunden zu verstehen? |  |
| 15 | …Informationen in den Medien darüber, wie Sie Ihren Gesundheitszustand verbessern können, zu verstehen? |  |
| 16 | …zu beurteilen, welche Alltagsgewohnheiten mit Ihrer Gesundheit zusammenhängen? |  |

Table 1: HLS-EU-Q16 German used in this study (1,6)

## Study Sample Size:

Sample size was determined a priori. The sample size was determined by the precision (i.e. length of the 95% confidence interval) of the correlation between the primary endpoint subjective emergency condition severity, separately assessed by patient, nurse, and physician. In the pilot trial there was a moderate correlation of Spearman rank correlation coefficient of 0.38, and 248 patients needed to be analyzed, so that the length of the 95% confidence interval of the correlation coefficient should not exceed 0.3. To account for a dropout rate of 20% 300-330 patients needed to be recruited.

## Biometrical Approach

Two a-priori hypotheses guided the analyses. Hypothesis 1 predicted that patients with limited health literacy (HL) would agree less with clinicians about the severity of their condition, whereas patients with adequate HL would agree more. Accordingly, **Spearman rank-order correlations (ρ)** were computed between the patient’s rating and each clinician’s rating for the full sample and separately within the inadequate-, problematic-, and adequate-HL groups. Fisher’s z values are reported to describe effect size and to compare correlation magnitudes across groups. Hypothesis 2 posited that limited HL would yield larger patient–clinician discrepancies, whereas adequate HL would yield smaller discrepancies. Signed and absolute discrepancy scores were therefore compared among the three HL groups with the **Kruskal–Wallis test**. Whenever the omnibus test was significant, pair-wise Dunn tests with Bonferroni-adjusted p values were carried out. Effect size for these group comparisons is provided as eta-squared (η²). Pre-analysis diagnostics included Shapiro–Wilk tests and Q–Q plots for univariate normality, and Levene’s test for homogeneity of variance. Because several variables deviated from normality while variances were acceptable, the non-parametric procedures noted above were retained. To examine whether HL predicts the **magnitude** of disagreement beyond demographics, multiple ordinary-least-squares (OLS) regressions were run with each absolute discrepancy score as the dependent variable and HL score, age, education, and gender (0 = female, 1 = male) entered simultaneously. Regression diagnostics (linearity, homoscedasticity, normality of residuals) were satisfactory. Unstandardized coefficients (B), robust standard errors, t values, R², and two-tailed p values (α = .05) are reported.

## Categories of diagnoses:

| **Diagnosis Severity Category** | **Examples** |
| --- | --- |
| **0**  Not threatening | No patients were assigned to this category |
| **1**  Barely threatening | - Swelling of a finger - Feet bruise - Small superficial cut of the skin |
| **2**  Possibly threatening | - Hypertension - Ankle joint sprain - Low-risk syncope |
| **3**  Threatening | - Urinary tract infection - Vertigo - Restlessness - Distal radius fracture |
| **4**  Possibly serious | - Anaemia - Abdominal pain - Supraventricular tachycardia |
| **5**  Serious | - Dyspnoea - Phlegmon - Erysipelas - Tibia fracture - Midface fracture |
| **6**  Very serious | - Pneumonia - Chest pain - Amaurosis fugax - Weber C fracture |
| **7**  Critical | - Stroke - Cholangitis - Acute confusion - Pelvic ring fracture |
| **8**  Likely life-threatening | - Odontoid fracture - Peripheral pulmonary embolism - Malignant stroke |
| **9**  Life-threatening | - Sepsis - Central pulmonary embolism |
| **10**  Immediately life-threatening | - Septic shock - Obstructive myocardial infarction |

Supplemental digital content 3: Diagnosis severity assessments by emergency medicine specialists with examples. Only examples that occurred in our study were listed. 0 points were not assigned.

## Diagnoses of included patients

The diagnoses of the included patients were distributed as expected. Abdominal and back pain were the most common (5%), followed by lower/upper limb injury, chest pain, and head trauma (3%). The ICD10 diagnosis and CEDIS presenting complaints used for treatment were also used for the analysis. The distribution of presenting complaints from the local ED according to the register for the entire year 2024 was as follows(9): abdominal pain (6%), back pain (5%), upper limb injury (4%), lower limb injury (3%), chest pain (3%), head trauma (3%).

Separated by the three HL levels, this distribution did not change. Abdominal pain, back pain, and chest pain were the most common (4%) diagnoses in patients with adequate HL. Abdominal pain, back pain, and lower limb injury were the most common (3-4%) diagnoses in patients with problematic HL. Abdominal pain, back pain, and head trauma were the most common (3-4%) diagnoses in patients with inadequate HL. The distribution of diagnoses in the included patients was representative.

## Covariate Adjustment Procedure:

Severity assessments were covariate-adjusted with a regression-based revisualization procedure. For each assessment (Patient, Nurse, Physician, Specialists) we estimated an OLS (Ordinary Least Squares) model across the full sample:

$$Rating\boldsymbol{=}\beta0+\beta1 \left( Age \right)+\beta2 \left( Educationi \right)+\beta3 (Gender)+\varepsilon$$

All three covariates were centered on their grand means. The individual adjustment term was subtracted from each raw score, yielding an adjusted assessment that represents the value a respondent would have shown if age, education, and gender were exactly average.

$$Adjustment\boldsymbol{=}\beta1 \left( Age-\bar{Age} \right)+\beta2 \left( Education-\bar{Education} \right)+\beta3 (Gender-\bar{Gender})$$

## Multiple regression model for patient-related data and Severe Outcome

| **Predictor** | *B (SE)* | *OR* | *95 % CI (OR)* | *p* |
| --- | --- | --- | --- | --- |
| Intercept | –6.79 (1.90) |  |  | < .001 |
| HLS-EU-Q16 score | –0.17 (0.05) | 0.84 | 0.75 – 0.93 | .001 |
| Patient assessment | 0.19 (0.06) | 1.21 | 1.07 – 1.37 | .003 |
| Age | –0.01 (0.01) | 0.99 | 0.97 – 1.01 | .225 |
| Education | –0.19 (0.08) | 0.83 | 0.70 – 0.98 | .025 |
| Gender | 0.18 (0.24) | 1.20 | 0.75 – 1.93 | .439 |

## Multiple regression model for Discrepancy

| **Predictor** | *Abs Disc All* | *Abs Disc Medical* | *Abs Disc Specialist* |
| --- | --- | --- | --- |
| Intercept | 7.12 (2.34)* | 5.93 (2.36)* | 8.96 (2.52)* |
| HLS-EU-Q16 score | –0.15 (0.06)* | –0.14 (0.06)* | –0.23 (0.06)* |
| Age | –0.02 (0.02) | –0.01 (0.02) | –0.04 (0.02)* |
| Education | –0.33 (0.22) | –0.25 (0.23) | –0.66 (0.25)* |
| Gender | –0.19 (0.23) | –0.01 (0.23) | –0.32 (0.25) |
| R^2^ | .04 | .02 | .07 |

Table 2: Unstandardized coefficients B (SE). *Asterisk marks significant effects.

| **Predictor** | *B (SE)* | *OR* | *95 % CI (OR)* | *p* |
| --- | --- | --- | --- | --- |
| Intercept | –9.43 (2.79) |  |  | < .001 |
| Age | –0.02 (0.01) | 0.98 | 0.96 – 1.00 | .063 |
| Gender | –0.11 (0.26) | 0.90 | 0.54 – 1.49 | .669 |
| Education | –0.08 (0.08) | 0.93 | 0.80 – 1.09 | .370 |
| Patient assessment | –0.05 (0.07) | 0.95 | 0.84 – 1.09 | .476 |
| Physician assessment | 0.20 (0.10) | 1.22 | 1.01 – 1.47 | .040 |
| Nurse assessment | 0.18 (0.09) | 1.20 | 1.01 – 1.43 | .037 |
| HLS-EU-Q16 score | –0.13 (0.06) | 0.88 | 0.78 – 0.99 | .028 |
| Discrepancy Medical Team | 0.26 (0.10) | 1.30 | 1.07 – 1.59 | .008 |

Table 3: Unstandardized coefficients B (SE). *Asterisk marks significant effects.

# References:

1. Sørensen K, Van Den Broucke S, Pelikan JM, Fullam J, Doyle G, Slonska Z, Kondilis B, Stoffels V, Osborne RH, Brand H. Measuring health literacy in populations: illuminating the design and development process of the European Health Literacy Survey Questionnaire (HLS-EU-Q). *BMC Public Health* (2013) 13:948. doi: 10.1186/1471-2458-13-948

2. Jordan S, Hoebel J. Gesundheitskompetenz von Erwachsenen in Deutschland: Ergebnisse der Studie „Gesundheit in Deutschland aktuell“ (GEDA). *Bundesgesundheitsblatt - Gesundheitsforschung - Gesundheitsschutz* (2015) 58:942–950. doi: 10.1007/s00103-015-2200-z

3. Sørensen K, Pelikan JM, Röthlin F, Ganahl K, Slonska Z, Doyle G, Fullam J, Kondilis B, Agrafiotis D, Uiters E, et al. Health literacy in Europe: comparative results of the European health literacy survey (HLS-EU). *Eur J Public Health* (2015) 25:1053–1058. doi: 10.1093/eurpub/ckv043

4. Sørensen K, Van Den Broucke S, Fullam J, Doyle G, Pelikan J, Slonska Z, Brand H, (HLS-EU) Consortium Health Literacy Project European. Health literacy and public health: A systematic review and integration of definitions and models. *BMC Public Health* (2012) 12:80. doi: 10.1186/1471-2458-12-80

5. Pelikan JM, Ganahl K, Van Den Broucke S, Sørensen K. “Measuring health literacy in Europe: Introducing the European Health Literacy Survey Questionnaire (HLS-EU-Q).,” In: Okan O, Bauer U, Levin-Zamir D, Pinheiro P, Sørensen K, editors. *International Handbook of Health Literacy*. Policy Press (2019). p. 115–138 doi: 10.51952/9781447344520.ch008

6. Röthlin F, Pelikan J, Ganahl K. Die Gesundheitskompetenz von 15-jährigen Jugendlichen in Österreich. (2013)

7. Pelikan JM, Ganahl K. Measuring Health Literacy in General Populations: Primary Findings from the HLS-EU Consortium’s Health Literacy Assessment Effort. *Stud Health Technol Inform* (2017) 240:34–59.

8. Pelikan JM, Straßmayr C, Link T, Miksova D, Nowak P, Griebler R, Dietscher C, Van Den Broucke S, Charafeddine R, Yanakieva A, et al. International Report on the Methodology, Results, and Recommendations of the European Health Literacy Population Survey 2019-2021 (HLS19) of M-POHL. (2021) doi: 10.13140/RG.2.2.27894.06721

9. AKTIN Research Group. Jahresbericht 2024 des AKTIN-Notaufnahmeregisters. (2025) doi: 10.24352/UB.OVGU-2025-086
